# Time spent by Belgian hospital pharmacists on supply disruptions and drug shortages: An exploratory study

**DOI:** 10.1371/journal.pone.0174556

**Published:** 2017-03-28

**Authors:** Elfi De Weerdt, Thomas De Rijdt, Steven Simoens, Minne Casteels, Isabelle Huys

**Affiliations:** 1 KU Leuven, Dept. Pharmaceutical and Pharmacological Sciences, Herestraat, Leuven, Belgium; 2 University Hospitals Leuven, Pharmacy department, UZ Herestraat, Leuven, Belgium; Jagiellonian University, POLAND

## Abstract

**Introduction:**

Supply problems of drugs are an increasing and worldwide problem, also in Belgium. Hospital pharmacists try to manage drug supply problems to minimize the impact on patient care. This study aims to quantify in a detailed manner how much time employees of 17 Belgian hospital pharmacies spend on drug supply problems.

**Methods:**

During six months, employees of Belgian hospital pharmacies filled in the daily time spent on drug supply problems using a template containing all steps which can be executed to manage drug supply problems. Additionally, Belgian hospital pharmacists were asked to report the drugs which experienced drug supply problems together with the solution for this problem.

**Results:**

Hospital pharmacists spent a median of 109 minutes a week on drug supply problems, with a minimum of 40 minutes per week and a maximum of 216 minutes per week. Fifty-nine percent of the total time spent on drug supply problems was executed by hospital pharmacists, 27% by pharmacy technicians; the rest was performed by logistic or administrative personnel. About one third of the total time spent was invested in gathering information on the supply problem. About two third of the supply disruptions caused drug shortages, meaning there was a need to switch to another (generic) therapeutic alternative. For most drug shortages, a Belgian generic medicine could be found. However in some cases, the alternative had to be ordered abroad or for some drug shortages, no alternative was available.

**Conclusion:**

These exploratory results on time spent by hospital pharmacists on drug supply problems in Belgium highlight the economic impact of drug supply problems for hospital pharmacies. A fully reliable, daily updated list on the federal agencies websites would be a major help to hospital pharmacists.

## Introduction

Drug supply problems are an increasing and worldwide problem [1–5]. Different definitions are used to define drug supply problems, also referred to as drug or medicine shortages [6]. From an economic point of view, a shortage is defined as: “when the supply does not meet the demand”. However the demand side can be considered at pharmacy level or at patient level. Diverse organizations adapted this general definition according to the interests of the organization [6]. In this paper, drug supply problems refer to supply disruptions (when the supply of drugs does not meet the demand at the level of pharmacies and wholesalers) and drug shortages (when the supply of drugs does not meet the demand at consumers/patients level).

Causes of drug supply problems are complex and multi-factorial [7, 8]. Quality and manufacturing problems, shortages of the active pharmaceutical ingredient (API) and an unforeseen demand have a large share in the known causes for supply problems [9, 10]. However, the real causes for supply disruptions and drug shortages remain often unknown [4, 9].

Drug shortages have an impact on every stakeholder [9, 11–16]. Supply disruptions and drug shortages cause reputational damage for the manufacturers. When manufacturers are unable to supply the market, they need to inform national competent authorities (i.e. agencies which are responsible for the regulation of human and veterinary medicinal products in the individual countries of the EU) [17, 18]. These national competent authorities invest time in updating national databases of supply problems and controlling information received from manufacturers [8, 19]. Pharmacists search for the best therapeutic alternative treatment for patients in consultation with prescribing health professionals [4, 20, 21]. These healthcare professionals need to change prescriptions towards drugs they are less familiar with [9, 22, 23]. The worst potential impact a supply problem might cause is considered at patient level. Drug substitution may lead amongst others to less effective therapy or more side effects. US literature documents patients even died due to drug shortages [9, 11, 24].

According to diverse surveys, hospital pharmacists spend a lot of time on managing supply disruptions and drug shortages [4, 5, 15, 21, 25, 26]. In US, hospital pharmacists spent about 9 hours per week on drug supply problems, while hospital technicians spent 8 hours per week [15]. European results range from only one hour per week to more than 15 hours per week [4]. A study performed by the University of Ghent estimated an average of 34.6 hours per week spent on supply problems with a minimum of 18.0 hours per week and a maximum of 51.5 hours per week [5]. However, as long as few data are available on the amount of time employees of hospital pharmacies spend on supply problems and consequently on the steps of managing supply disruptions and drug shortages, it remains hard to develop measures to mitigate the problem [15, 16]. The main objective of this study is to quantify the time employees of Belgian hospital pharmacies spend on drug supply problems. Second, this study investigates who spends the most time on managing drug supply problems and which steps of managing drug supply problems were most time-consuming. Based on the results, recommendations will be formulated to mitigate the problem. Additionally, the study aims to identify the actions hospital pharmacists undertook to minimize patient’s impact. Because drug supply problems vary over time, the study ran over a period of 6 months.

## Methods

### Study design

To assess the time spent on supply problems of medicines by Belgian hospital pharmacies, a time and motion study was set up [27]. This is a non-interventional, descriptive study whereby employees of Belgian hospital pharmacies were requested to note all the time they spent on managing supply disruptions and drug shortages. The ethics committee of the University Hospitals Leuven was consulted, but for this study design (a study of public behaviour of the staff of hospital pharmacies that is purely observational and non-interventional) no ethical approval was needed. A waiver from the ethics committee of the University Hospitals Leuven was received.

In Belgium, hospital pharmacies mainly order their medicines directly from manufacturers. A small part is ordered at wholesalers. If the product experience supply problems, hospital pharmacists will search for generic medicines or alternative medicines by other manufacturers or wholesalers. If the Belgian market has no therapeutic alternatives available, hospital pharmacists will try to order a therapeutic alternative abroad. Foreign therapeutic alternative medicines are not always considered for reimbursement.

In collaboration with a Flemish hospital pharmacist, a template in Dutch language was developed covering the most relevant steps an employee of a hospital pharmacy can undertake to manage drug supply problems, based on the supply chain. A first version of the template was sent to twelve randomly selected Flemish hospital pharmacists for validation. Minor adjustments to the template were made based on suggestions of the hospital pharmacists and consensus among them was reached on the final version of the template. Afterwards, the template was translated in French by a bilingual hospital pharmacist. To control for potential language errors, three randomly selected Walloon hospital pharmacists validated the French template. Once again consensus was reached. An English version of the template is displayed in S1 Table. The template was divided in three main sections. The first section contained the steps related to the moment a hospital pharmacist became aware of a supply disruption and the way they intended to overcome the supply disruption or drug shortage. The second section covered steps associated with the arrival of the alternative medicine and the third section was linked to the follow-up of supply problems.

The time spent on drug supply problems was measured. As mentioned in the introduction, in this paper the wordings “drug supply problems” were used, which covers drug supply disruptions and drug shortages. A drug supply disruption was defined as ‘a disruption of the normal supply of a drug’. However, this disruption did not necessary lead to a drug shortage in the hospital. A drug shortage was defined as ‘a supply problem which leads to changing the standard therapy into an alternative therapy’. This distinction was based upon the fact that hospital pharmacists do not know whether a supply disruption will result in a drug shortage.

Templates for daily reporting during the study period were bundled in monthly booklets, including overlapping periods. To ensure all time spent on drug supply problems was captured by the participants, multiple booklets were distributed across the hospital pharmacy and its divisions. The amount of booklets distributed depended on the size of the hospital pharmacy and its employees. The template needed to be filled in daily with the total time spent on supply problems, hence not specific per supply problem.

### Data collection

All Belgian hospital pharmacists were invited to confirm their participation via email sent by the Flemish and Walloon professional organizations of hospital pharmacists (resp. Flemish Association of Hospital Pharmacists and the Francophone Association of the Belgium hospital pharmacists). The invitation and a reminder were sent in February and March 2015, respectively. The study started on April 1^st^, 2015 and ended on October 4^th^, 2015.

Respondents were contacted for a kick-off meeting before the start of the study for the provision of further information about the study and to deliver the booklets for the first month (April 2015). Each month, a new appointment was made to collect the booklets of the previous month and to bring new booklets for the upcoming month. From these monthly visits, the authors availed to remind the hospital pharmacists to fill in the templates. Hospital pharmacists contributed voluntarily and were not remunerated.

As the time and motion study considered the time spent on all supply problems, it did not take into account the exact number of supply disruptions and/or drug shortages. Therefore a second booklet was developed to collect background information about drugs experiencing supply problems (S2 Table). Hospital pharmacists were asked to fill in these booklets as detailed as possible. Information was gathered regarding whether or not the supply disruption caused a drug shortage and what action the hospital pharmacist performed to solve the drug shortage.

### Data validation

Once all the data were inserted in a database (Microsoft Excel 2013), the data of the participants were inspected. If the authors suspected the templates were not filled in correctly based on barely filled in templates, hospital pharmacists were contacted. Data from participants were excluded if they admitted or self-reported they were not able to fill in the template correctly during a certain period (at least three consecutive weeks).

The preliminary results were presented to the employees of all the hospital pharmacies who participated to validate the observed results and trends. This validation meeting was also used to discuss potential solutions to reduce the time spent on supply problems of drugs.

### Data analyses

Mainly descriptive analyses were performed due to the small sample size, using Excel 2013 and IBM SPSS Statistics 23.The total time spent per week was investigated, as well as the time spent on each step undertaken to manage supply problems and the time spent by employees of the hospital pharmacies. Results were displayed in minutes and relative numbers were presented as percentages. The considered sample size (N) was included in the result.

To check whether the time spent by hospital pharmacists was correlated to the number of beds or the number of employees, the Pearson’s correlation was calculated using IBM SPSS Statistics 23.

From the background information papers, the type and number of supply disruptions and drug shortages were analysed, together with the solutions for drug shortages. Only supply problems for which the question ‘whether or not the supply disruption caused a drug shortage’ was answered, were considered for further analyses. Supply problems were reported according to the WHO ATC-system [28].

## Results

### Time spent of hospital pharmacies

#### Sample characteristics

From the 146 hospitals in Belgium which were invited to participate (counting 93 general, seven university, 35 psychiatric and 11 specialized hospitals), 22 hospitals started the study (about 15%), including 17 general, three university and two psychiatric hospitals. Apart from the two psychiatric hospitals, no specialized hospitals responded and hence only these two were included in the study. Five hospital pharmacies were excluded based on the exclusion criteria. Thus eventually, 17 hospitals (13 general, two university and two psychiatric hospitals) were retained (about 12%) for further analysis.

Ten of the included hospitals started the study on April 1^st^, 2015. Due to late responses of some hospitals, the authors decided to start a second group of six hospitals on April 27^th^, 2015. One hospital was allowed to start on May 25^th^, 2015 to improve the representativeness of the study. Our sample (22 hospitals) and the eventually 17 included hospital pharmacies are representative sample of the Belgian situation in terms of the geographical situation. In terms of the type of hospitals, an underrepresentation is observed for psychiatric hospitals and an overrepresentation for university hospitals.

The described study period includes 27 weeks. However, as more than half of the hospitals did not fill in the templates during the last week, the time spent of the last week was not considered in the data analyses. During the validation session, hospital pharmacists admitted the study period was long and that at the end personnel of the pharmacies lacked filling in the templates.

Hospital pharmacies were divided in two groups: (i) general and university hospitals and (ii) psychiatric hospitals. This division was based on the difference in the type of medicines used by particular hospital pharmacies.

#### Time spent on supply problems by general and university hospital pharmacies

**Fig 1:**
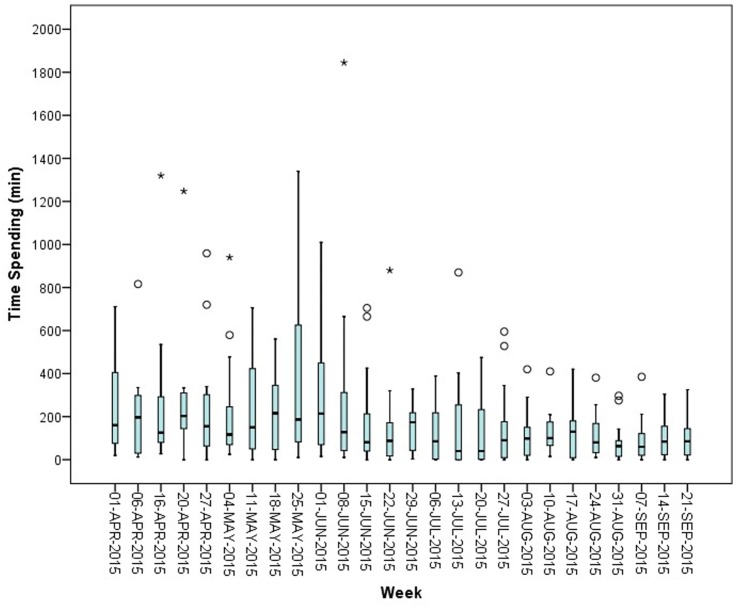
Median (IQR) time spent (in min) per week on supply problems by general and university hospitals in Belgium (N = 15). ° = outlier [1.5 IQR– 3 IQR]; * = extreme (> 3 IQR).

Fig 1 displays the median time spent in the general and university hospitals (N = 15), together with its interquartile range (IQR) over the period of 26 weeks. Medians and interquartile ranges were used to express the variables, as the variables were not normally distributed. Based on our results, the median time spent on supply problems is 109 minutes a week, with a minimum of 40 minutes per week and a maximum of 216 minutes per week.

The time spent during the summer months (July, August and September 2016) is lower than during the months before the summer (April, May and June 2016), namely a median of 85 minutes per week and 155 minutes per week respectively. After data validation with the hospital pharmacists, two reasons for this difference were mentioned. First, hospital pharmacists indicated that during the summer, fewer patients were hospitalized, resulting into quiet periods in the hospitals. Secondly, holiday periods of hospital pharmacy employees during the summer lead to discontinuations in completing the templates during this period. Therefore no further statistical analyses were performed on the data to explore this specific difference in time spent across these months.

**Fig 2:**
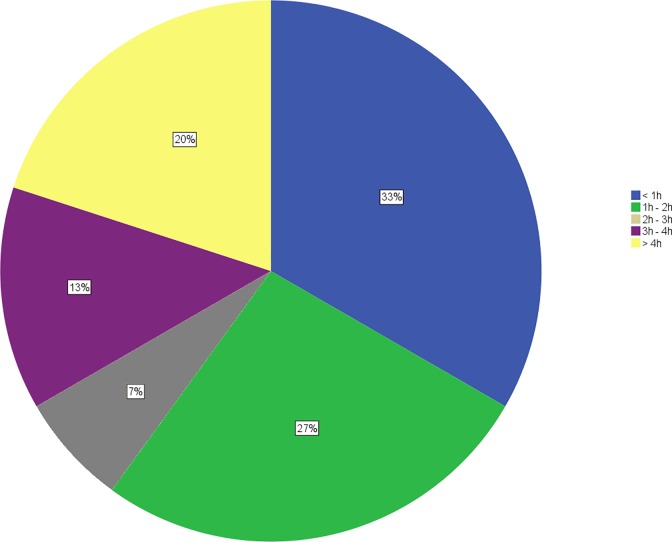
Distribution of general and university hospital pharmacies based on the median time spent on supply problems in hours per week (N = 15).

The outliers in Fig 1 can be attributed to the major differences across hospital pharmacies and the time spent on supply problems of drugs. Fig 2 displays the distribution of the average time spent per week by the hospital pharmacies (N = 15). One third of hospital pharmacists spent less than one hour a week, yet an equal number of hospital pharmacies spent more than three hours a week on managing supply disruptions and drug shortages. The number of hospital beds or the number of employees in the hospital pharmacies were not related to the time spent on drug supply problems (p > 0.05). However, during the validation session, stock management was suggested as a potential confounding factor. A hospital with a high inventory level would experience fewer supply problems compared to a hospital pharmacy with a low inventory level. This was also observed in a recently relocated hospital pharmacy, whose stock, before the study, was increased due to the movement. This hospital spent a median of less than one hour on supply problems, which might confirm stock management as confounding factor on the time spent on supply problems.

**Fig 3:**
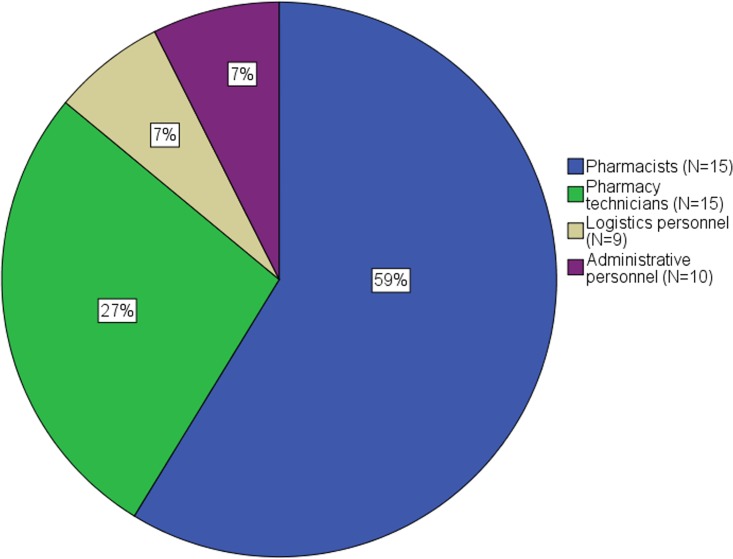
Distribution of total time spent on managing supply disruptions and drug shortages by the employees of general and university hospital pharmacies (N = 15).

Most work to manage supply disruptions and drug shortages was performed by the hospital pharmacists themselves, representing about 60% of the total time spent on supply problems. One fourth of the total time spent on supply problems was performed by the pharmacy technicians. The remaining time spent was executed by logistics and administrative personnel, however, as displayed in Fig 3, not every hospital employed logistics and administrative personnel in the hospital pharmacy. Considering the median of 109 minutes spent on drug supply problems by hospital pharmacies, hospital pharmacists spent about one hour on supply disruptions and drug shortages, pharmacy technicians about half an hour and logistics and administrative personnel each about 10 minutes.

**Fig 4:**
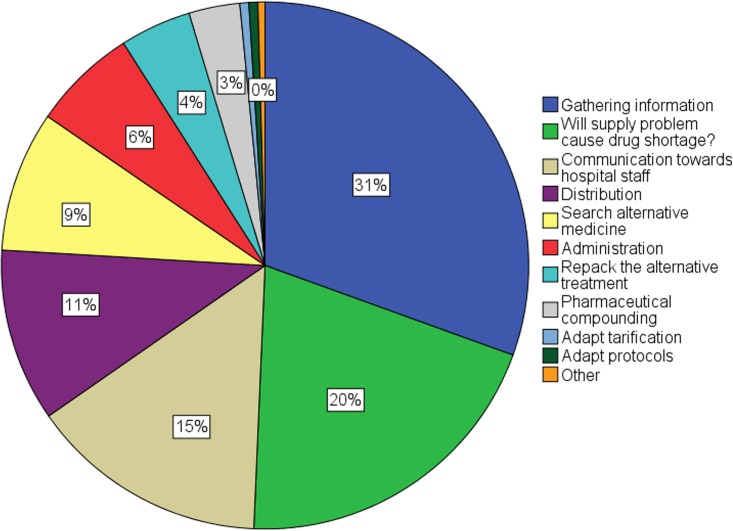
Time spent (% of total time spent) on steps to manage supply problems by general and university hospital pharmacies (N = 15).

Considering the different steps in managing supply problems, employees of hospital pharmacies spent the most time on gathering information about supply disruptions (Fig 4). This step included contacting the supplier, the manufacturers, other campuses or other hospitals to get more information about the supply disruption, but also the follow-up of this information. The gathering of information represented about a quarter of the total time spent on supply problems. The second most time consuming step was to control whether the supply disruption will cause a drug shortage at the hospital. The employees of the hospital needed to inspect whether the remaining stock (in the hospital pharmacy and decentrally, in the departments of the hospital) was sufficient to overcome the period of no delivery. Other labour-intensive steps were communication towards hospital employees and “distribution”. “Distribution” involved the distribution of the alternative treatment as well as the distribution of the usual drug once back in stock. Other steps, which were less time consuming, were the search for alternative treatments, administration that was associated with ordering the alternative drug and the usual drug, repacking the alternative treatment, pharmaceutical compounding and adapting tarification and protocols.

**Fig 5:**
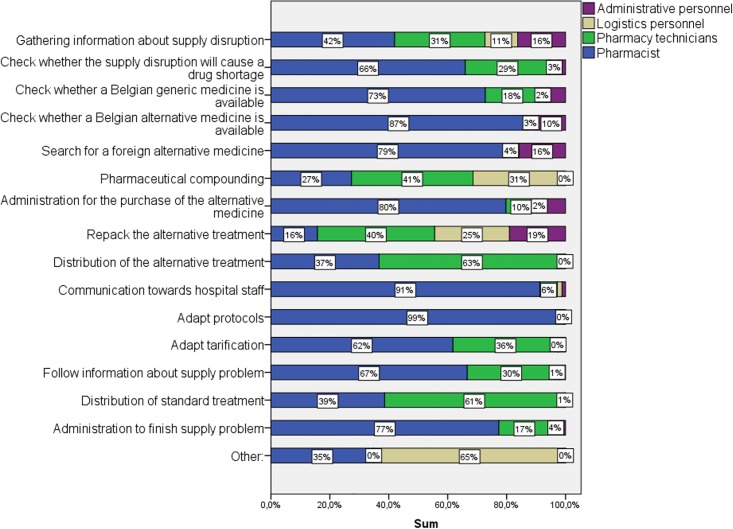
Percentage of time spent by general and university hospital pharmacy employees per step for managing drug shortages (N = 15).

Fig 5 displays the different steps together with the percentage of the total time spent of the employees of the hospital pharmacy. Hospital pharmacists were almost fully active in the adaptation of protocols (i.e. changing the electronic system), the communication towards other hospital staff and the administration associated with drug supply problems. During the validation session, all participants agreed that these steps belong to the hospital pharmacists’ job responsibilities. However, according to our study, hospital pharmacists spent also a lot of time on searching for alternative treatments. Most of the hospital pharmacists agreed that the search for alternative medicines could be performed by hospital technicians. However, the final decision about the potential alternative treatment should be made by the hospital pharmacist.

#### Time spent on drug supply problems at psychiatric hospitals

Only 2 psychiatric hospitals participated in the study. The median time spent on supply problems of medicines by psychiatric hospitals was only six minutes per week. Of the total time spent on supply problems, 71% was executed by the hospital pharmacists and the remaining 29% was performed by the pharmacy technicians. Psychiatric hospitals also spent most time on gathering information about supply disruptions, again followed by “check whether the supply disruption will cause a drug shortage”. Both steps occupy about one third of the total time spent on supply problems of medicines.

### Reported supply problems

Additional to the time spent of hospital pharmacies, we investigated the number of supply disruptions, how often supply disruptions turned into drug shortages, how supply problems were communicated and in case the supply disruption caused a drug shortage, how it was solved.

From the 17 included hospitals, one hospital did not fill in the information templates about the supply problems. It should be stated that hospital pharmacists mentioned they did not fill in the information papers for every supply disruption. The following data are therefore based on the reported supply problems of 16 hospitals.

**Fig 6:**
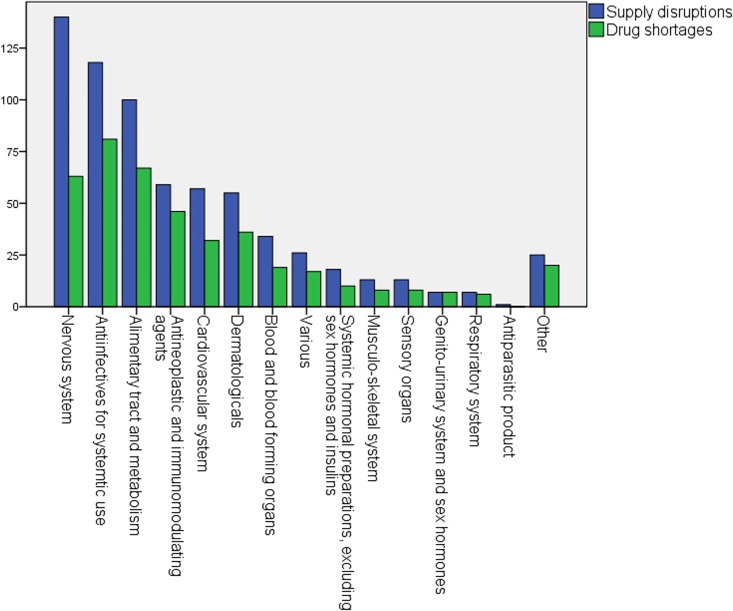
Number of supply disruptions and drug shortages according to the first level of the ATC-code. Other = drugs which are not assigned to an ATC-code

A total number of 673 supply disruptions were reported during the study period of 26 weeks. Hospital pharmacists were questioned whether the supply disruption turned out in a drug shortage or if they managed the supply problem by adapting guidelines for the use of drugs which were in short supply. About two thirds (62%) of the supply disruptions caused drug shortages (N = 420) (Fig 6). For 37% of the supply disruptions, sufficient stock avoided a drug shortage. ‘Adopting guidelines for the use of drugs which were in short supply’ accounted for only 1% of the supply disruptions, which did not cause drug shortages.

Fig 6 displays the ATC categories where supply disruptions and drug shortages were situated. During the study period, supply disruptions were mostly experienced for drugs classified for the treatment of the nervous system, especially analgesics (N02) and psycholeptics (N05) were affected. Next, anti-infective medicines for systematic use were mostly affected, with anti-bacterial medicines (J01) experiencing the most disruptions. Drugs used for the treatment of the alimentary tract and metabolism (A), antineoplastic and immunomodulating agents (L) and cardiovascular diseases (C) also experienced often drug supply disruptions. Most affected drug subclasses of the latter were drugs for functional gastrointestinal disorders (A03), antineoplastic agents (L01) and drugs for cardiac therapy (C01) and diuretics (C03).

Drug shortages, on the other hand, mostly affected anti-infectives for systematic use (J), especially the subclass of anti-bacterials (J01). Secondly drugs for the treatment of the alimentary tract and metabolism (A) encountered often drug shortages, mainly alizapride and metoclopramide (A03). Though drugs for the nervous system (N) experienced the most supply disruptions took third place, less than half of them turned into drug shortages. Antineoplastic and immunomodulating agents, especially antineoplastic drugs (L01) and dermatological drugs (D), with mainly shortages of corticosteroids (D07), complemented the top five of most affected drug shortages (Fig 6).

For about two thirds of the drug shortages (N = 420), a generic medicine (= same API) solved the problem. For one fifth of the shortages, the drug was replaced by an alternative medicine (= other API) and for 8% of the drug shortages no alternative was found (64% = same API; 19% = different API; 9% = no alternative; 9% = no alternative). Solutions for drug shortages (N = 420) were mainly domestic, while for about one tenth of the drug shortages a foreign solution was found (59% = domestic solution; 13% = foreign solution; 28% = blanks).

## Discussion

Pharmacies of general and university hospitals spent about two hours per week on supply disruptions and drug shortages. Though this seems not very much, large differences were observed between hospitals. In addition, the two hours are spread over a whole week, leading to multiple interruptions of pharmacists’ activities, which might create the impression of a continuous involvement in drug supply management in practice. Hospital pharmacists accounted for about 60% of the total time spent. The step “gathering information about the supply disruption” was responsible for about one third of the total time spent on supply disruptions and drug shortages. Psychiatric hospital pharmacies spent much less time on supply problems compared to the general and university hospital pharmacies, i.e. six minutes per week. In total 673 supply problems were reported through the information sheets. Two thirds of these reported supply disruptions turned into drug shortages. Most of them were managed by substituting by a domestic generic drug.

The median time spent observed per hospital in this study (± two hours a week) is much lower than the previously reported time spent of hospital pharmacies on supply problems [4, 5, 15, 25, 26]. According to the European association of hospital pharmacists (EAHP), the time spent was measured through a survey and ranges between one hour a week and 15 hours a week [4]. This was similar with results obtained in the US [15]. A study of the university hospital in Ghent estimated the time spent on supply problems around 35 hours per week [5]. One explanation for the difference with the study of Ghent can be assigned to the type and number of hospitals. The study of Ghent considered only the time spent of two hospital pharmacies (one university hospital pharmacy and one general hospital pharmacy) [5]. Our study considered 15 general and university hospital pharmacies. Another explanation for the observed differences with the EAHP study and the study of Ghent can be allocated to the used methodology. For the survey of EAHP it remains unclear how hospital pharmacists interpreted “time spent on medicine shortages”. Did the question cover the time spent by all employees as well as every step of the management of supply problems? The study of Ghent, on the other hand, did cover all steps in the management of supply disruptions and drug shortages and considered all employees of the hospital pharmacies who are burdened with supply problems, as in our study. However, the estimated 35 hours per week by Claus *et al*. was the result of answering the question: “how many hours per week do you spend on managing drug shortages?” [5]. This implies a retrospective subjective answer of employees of two hospital pharmacies. Our study used “self-reporting” methodology and included different hospitals, which leads to more objective results [29].

Gathering information about the supply disruption represents a substantial share of the total time spent on supply problems of drugs (about 25%). This large share is devoted to the fact that every hospital pharmacist is performing the same actions to explore the cause and the duration of the supply problem. It should be noted that multiple hospitals have often the same supplier and thus hospital pharmacists are doing the same steps as their colleagues. In addition, pharmacists in hospitals are paid by the government [30]. Therefore, actions should be taken to improve this communication failure.

Once hospital pharmacists are aware of the threat of a drug shortage, they start looking for alternative treatments often from other manufacturers. If several hospital pharmacists order the same alternative treatment from the same manufacturer, it can occur the latter manufacturer cannot meet the sudden increased demand, resulting in a domino-effect of supply problems with different suppliers.

Our results indicated a calmer period (i.e. less supply problems) during summer months and this was confirmed during the validation session. During the summer months, less supply problems are experienced by the hospital pharmacists, which is probably correlated to the reduction of elective surgeries during these months. In the months November and December hospital pharmacists appear to encounter more supply disruptions and drug shortages, probably due to lower inventories at the manufacturers’ sites devoted to the annual report. A second potential reason can be found due to the fact that many manufacturers close during the Christmas holidays.

Stock management plays an important role in causing or preventing drug shortages. As discussed above, a higher stock at hospital pharmacies can help to overcome short-term supply disruptions. Keeping a buffer and a well-managed stock is also important to avoid managed inventory shortages [26]. This was confirmed by hospital pharmacists in the validation session. They argue that hospitals with an inventory of one week will experience more supply problems compared to hospital with a stock of one month. Participating hospital pharmacists became more aware of the importance of stock management and therefore mentioned during the validation session that they considered changing their internal procedures in the future to improve the management of supply problems. Additionally, the moment of ordering has also an impact on causing drug shortages, especially for short-term supply disruptions. On the other hand a right balance should be found in order not to unnecessarily increase stocks, which may increase the supply problems on the market.

This study design has several advantages. Self-reporting easily allows the participation of multiple hospital pharmacies [29, 31]. A second advantage of our study is that the time spent on supply problems was measured over an extensive study period of 26 weeks. Thirdly, the results are based on the time spent by 17 hospital pharmacies, including general, university and psychiatric hospitals. Last, due to the detailed manner of investigating the time spent on drug supply problems, targeted measures can now be taken by several stakeholders to mitigate the problem.

### Study limitations

However, the study design has several limitations. Five hospital pharmacies were excluded because they admitted were not able to fill in the template during several weeks. It could be that also other hospitals experience likewise problems, but did not admit. This may lead to an underestimation of the total time spent on drug supply problems.

Another limitation of this study could be that participants can experience their involvement as troublesome and time-consuming. This might also lead to underreporting, especially when participants are overloaded with work [29, 31]. During the validation session, participants admitted they sometimes forgot to fill in the templates correctly and therefore our result will be an underestimation of the actual time spent. As discussed in the validation session, hospital pharmacists believe the time spent will be about half an hour more (about 2.5 hours per week).

A third disadvantage can be attributed to semantics. Participants might interpret the wording in the template differently, which is another disadvantage of the used method [31]. This will lead to inaccurate reporting of the time spent between the different steps, mentioned on the template; still the total time spent will not be influenced.

It could be argued that participating hospital pharmacies became more aware of handling drug supply problems and that during the study they changed their habits to manage the problem more efficiently. However changing their stock management cannot be achieved immediately since manufacturers should be able to react on the suddenly increased demand.

Also, the economic impact of supply problems was restricted in our study to the time spent by hospital pharmacists on supply disruptions and drug shortages, but also includes other factors such as increased costs for patients due to e.g. not-reimbursed treatments. Public pharmacies and manufacturers are also economically affected. These other aspects need to be considered in future research.

Supply problems of drugs are just a part of the supply problems where hospital pharmacists are confronted with. Supply problems of medical equipment and parenteral nutrition burden hospital pharmacists and technicians as well [32, 33]. These supply problems seem less obvious, still are important to be aware of and to follow up the magnitude of the problem.

### Potential solutions

One third of total time was spent on gathering “information about the supply problem”. This means that once there is a supply disruption, every hospital pharmacist (with the same manufacturer) needs to contact the manufacturer to find out what the exact problem is and how long the problem will last. However, this time spent can be easily reduced if manufacturers inform hospital pharmacies with an email or a letter accompanying the order explaining why there is a supply problem, how long it will last (or a time indication) and, if possible, suggesting potential alternatives. This will help both hospital pharmacies and manufacturers, since every hospital pharmacist undertakes the same actions and the manufacturer will have to answer the same questions.

The federal agency of medicines and health products (FAMHP) publishes daily a list of drug shortages on their website. Belgium is not the only country who publishes a list, other European countries as well as US update a list of drug shortages [19, 34–38]. However, the participating hospital pharmacists observe that the list is not always up to date. Especially during Christmas holidays, while most supply problems are experienced, the list of shortages fails. A fully reliable, every day updated list on the FAMHP website would be of major help to hospital pharmacists. Additionally, facilitating the information flow between the agencies and the hospital pharmacies can also be investigated, e.g. how is it done in the US [35, 36].

Secondary, manufacturers often do not meet the demand of the hospital pharmacists (partially delivered orders). Hospital pharmacists remains clueless whether the incomplete order is by mistake or due to supply problems. In case of supply problems, the manufacturer is not obliged to report since he is able to “deliver”. The Flemish Association of Hospital Pharmacists (VZA) uses a forum to communicate supply problems. However, hospital pharmacists believe this forum is not convenient and it is not the responsibility of hospital pharmacists to inform each other about supply problems.

As cited in the “results” section, a potential solution was suggested by some hospital pharmacists during the validation session, namely to give more responsibilities to pharmacy technicians in order to reduce the time spent of hospital pharmacists on supply problems. However, not every hospital pharmacy agrees with this solution. Some believe their pharmacy technicians are already occupied by their own job responsibilities and adding “searching for alternatives” would not reduce the total time spent.

## Conclusion

This time-and-motion study provided exploratory results on the time spent by hospital pharmacists on supply disruptions and drug shortages in a relatively small but representative sample of hospitals, showing a large variation in estimates of time spent. Further studies need to validate our results in a larger number of hospitals. However, our findings suggest that improvement of the communication between the different stakeholders can already reduce the total time spent by employees of hospital pharmacies. Therefore, a fully reliable, daily updated list on the website of the national competent authority (FAMHP in Belgium) would be a major help to hospital pharmacists, and this would be an easily achievable realistic recommendation to the FAHMP.

## Supporting information

S1 Table Template to report time spent on drug shortages. (DOCX)

S2 Table Information paper. (DOCX)

S1 Dataset Dataset on time spent by hospital pharmacies. (XLSX)

S2 Dataset Dataset on reported drug supply problems by participating hospital pharmacies. (XLSX)
