# Supplementary material for: Time spent by Belgian hospital pharmacists on supply disruptions and drug shortages: An exploratory study
Source: PLoS One. 2017 Mar 28;12(3):e0174556. doi: 10.1371/journal.pone.0174556 (PMC5370124; doi:10.1371/journal.pone.0174556)
Supplement: S1 Table — (DOCX) [file pone.0174556.s001.docx]

S1 Table: Template to report time spent on supply problems

|  | Pharmacist  (min) | Pharmacy technicians  (min) | Logistician  (min) | Administrative assistant  (min) |
| --- | --- | --- | --- | --- |
| Gathering information about supply disruption |  |  |  |  |
| Check whether supply disruption will cause a drug shortage |  |  |  |  |
| Check whether a Belgian generic medicine is available |  |  |  |  |
| Check whether a Belgian alternative medicine is available |  |  |  |  |
| Search for a foreign alternative treatment |  |  |  |  |
| Pharmaceutical compounding |  |  |  |  |
| Administration for the purchase of the alternative treatment |  |  |  |  |
| Repack the alternative treatment |  |  |  |  |
| Distribution of the alternative treatment |  |  |  |  |
| Communication towards hospital staff |  |  |  |  |
| Adapt protocols |  |  |  |  |
| Adapt tarification |  |  |  |  |
| Follow information of supply problem |  |  |  |  |
| Distribution of standard treatment |  |  |  |  |
| Administration to finish supply disruption/drug shortage |  |  |  |  |
| Other:………………………… |  |  |  |  |
