# Supplementary material for: Time spent by Belgian hospital pharmacists on supply disruptions and drug shortages: An exploratory study
Source: PLoS One. 2017 Mar 28;12(3):e0174556. doi: 10.1371/journal.pone.0174556 (PMC5370124; doi:10.1371/journal.pone.0174556)
Supplement: S2 Table — (DOCX) [file pone.0174556.s002.docx]

S2 Table: Information paper

| Which medicine experienced a potential drug shortage? | | | | | | |
| --- | --- | --- | --- | --- | --- | --- |
| Name | ……………………………………………………………………………….. | | | | | |
| Dose | ……………………………………………………………………………….. | | | | | |
| Package | ……………………………………………………………………………….. | | | | | |
| Company | ……………………………………………………………………………….. | | | | | |
| How is the supply disruption communicated? | | | | | | |
| Manufacturer | Other hospital | | | Order is not delivered | | Other: …………. |
| Did the supply disruption ended up in a drug shortage? | | | | | | |
| Yes | | | No, sufficient stock | | No, guidelines adopted | |
| If yes, what was the alternative? | | | | | | |
| Interior | | | | Foreign | |  |
| Generic medicine: | | ……………………………………………….. | | | | |
| Alternative medicine: | | ……………………………………………….. | | | | |
| No alternative | |  | | | |  |
